# Integrated Vegetative and Reproductive Traits Reveal Functional Groups and Assembly Mechanisms in a Subtropical Forest Ecotone

**DOI:** 10.3390/plants15030406

**Published:** 2026-01-29

**Authors:** Chenxing Xu, Lan Jiang, Jing Zhu, Xin We, Jinfu Liu, Daowei Xu, Zhaopeng Zhang, Xiangyi Guo, Zhongsheng He

**Affiliations:** 1 College of Forestry, Fujian Agriculture and Forestry University, Fuzhou 350002, China; xuchenxing1230xcx@163.com (C.X.); jlnaruto0629@126.com (L.J.); hdly0718@126.com (J.Z.); weiixn@163.com (X.W.); fjljf@fafu.edu.cn (J.L.); xudaowei2004446@126.com (D.X.); 2 College of Urban and Rural Construction (College of Rural Revitalization), Fuyang Institute of Technology, Fuyang 236000, China; 3 Department of Forestry, Xinyang Agriculture and Forestry University, Xinyang 464000, China; 4 Fujian Niumulin National Nature Reserve Administration, Quanzhou 362600, China; nmlbgs@163.com (Z.Z.); nmlgxy@163.com (X.G.)

**Keywords:** ecotone, functional traits, trait syndromes, woody plants

## Abstract

In species-rich forests, the integration of vegetative and reproductive traits defines plant ecological strategies and underpins community assembly. How these trait syndromes assemble into functional groups to facilitate species coexistence in ecotones remains unclear. To address this, we measured 17 key functional traits in 121 woody plant species, covering vegetative and reproductive traits, and used hierarchical clustering to classify these species into functional groups (FGs). We found the following: (1) The woody plant community exhibits distinct trait syndromes adapted to the ecotonal environment: evergreen species accounted for 84.3%, microphanerophytes dominated (95.04%), simple leaves and alternate phyllotaxy prevailed, and animal-mediated pollination (91.74%) and seed dispersal (77.69%) were the primary reproductive strategies. (2) The 121 species were classified into 10 optimal FGs based on integrated differences in vegetative traits (e.g., leaf morphology, life form, phyllotaxy) and reproductive traits (e.g., pollination/dispersal mode, inflorescence/fruit type). Most FGs were dominated by evergreen microphanerophytes, reflecting convergent adaptation to the subtropical ecotonal environment, while distinct adaptive strategies differentiated the groups: FG1 (solely *Meliosma rigida*) was distinguished by whorled phyllotaxy and large leaves, a specialization for high-light microhabitats; FG5, a unique deciduous group, comprised species (e.g., *Nyssa sinensis*) with alternate leaves and axillary inflorescences, adapting to seasonal resource fluctuations. (3) These FGs reflected adaptive strategies to diverse microhabitats: rare species in FG4 (e.g., *Acer cordatum*) adopted wind-dependent pollination/dispersal to cope with mountainous wind variability, while FGs 3, 7, 8, 10 relied on animal mutualism to ensure reproductive success, highlighting the role of plant–animal interactions in community structure. Our study clarifies the trait differentiation patterns and FG assembly mechanisms of woody plants in the mid-subtropical–south-subtropical ecotone. The integrated trait-based FG classification could provide insights into how species coexist via niche differentiation and offer a theoretical basis for biodiversity and ecosystem conservation.

## 1. Introduction

Understanding how plant species adapt to their environment is a central question in ecology. Functional traits—quantifiable attributes shaped through long-term evolution—serve as a key link between individual plant adaptation strategies and ecosystem functions [1]. These traits are typically categorized into vegetative traits (e.g., growth form, life form, leaf morphology) and reproductive traits (e.g., sexual system, propagule type, pollination and seed dispersal modes), which together determine a species’ ability to acquire resources, reproduce successfully, and respond to environmental fluctuations [2,3]. For instance, leaf traits such as leaf size and texture influence light capture and water use efficiency [4], while reproductive traits like dispersal mode regulate gene flow and regeneration capacity [5]. Importantly, trait syndromes such as animal-mediated pollination and seed dispersal reflect intricate plant–animal interactions that shape community dynamics and ecosystem resilience. Specifically, trait syndromes refer to adaptive combinations of functionally related traits at the species level, representing the core strategic expression of plants in response to the environment; functional groups (FGs), by contrast, are assemblages of species with convergent trait syndromes, forming a hierarchical relationship of “species strategy—community aggregation” [6].

To simplify the complexity of species-rich ecosystems, ecologists often classify plants into functional groups (FGs)—assemblages of species sharing similar trait syndromes and ecological roles [6]. This approach has become a powerful tool for elucidating community assembly, predicting ecosystem responses, and generalizing patterns across biomes. Globally, trait-based clustering has revealed adaptive strategies in diverse forest types, with climatic zones being one of the key correlative factors: tropical forests often group species by shade tolerance and dispersal syndromes [7], while temperate forests emphasize leaf phenology (deciduous vs. evergreen) and nutrient use efficiency [8]. It is important to note that climatic zones are not the sole criterion for such classification, as forest species compositions are inherently complex and shaped by multiple environmental factors (e.g., topography, microclimate, and biotic interactions). In transitional climates such as subtropical regions, trait trade-offs become more complex, and recent studies increasingly advocate for integrating both vegetative and reproductive traits to better capture functional diversity and niche partitioning [9,10]. Such integration is especially critical for detecting niche partitioning mechanisms that underpin species coexistence.

However, most existing studies still focus on a single trait dimension—either vegetative or reproductive—limiting our understanding of how plants coordinate multiple traits to adapt to heterogeneous environments [11,12]. This gap is particularly pronounced in ecotones, transitional zones between major biomes, which harbor high biodiversity and exhibit strong sensitivity to climate change [13,14]. Ecotonal communities often display unique trait combinations reflecting adaptations to fluctuating microhabitats, yet their functional group structures remain poorly characterized. In China, while numerous studies have characterized functional traits in core subtropical forests [15,16], a comprehensive, integrated trait-based classification of woody plants in ecotonal regions has received far less attention. Phyllotaxy is a fundamental architectural trait that directly determines light interception efficiency and resource partitioning among individuals [17], regulating leaf arrangement to minimize self-shading or maximize light capture in specific microenvironments [18]. Its variation across forest species is a key manifestation of adaptive strategy differentiation, enabling plants to occupy distinct light niches (e.g., understory vs. canopy) [19]. Leaf structure is prioritized in functional trait studies due to its direct links to plant metabolism, resource acquisition, and stress tolerance [4]. Traits such as leaf texture, size, margin type, and indumentum collectively define a species position along the leaf economic spectrum, reflecting trade-offs between growth rate and resource conservation [4]. This renders leaf structure a powerful tool for quantifying functional diversity and predicting community responses to environmental change. For instance, leathery leaves with thick cuticles enhance tolerance to drought and high irradiance, while papery leaves optimize photosynthetic efficiency in shaded and humid habitats [20]. Reproductive traits are crucial for functional classification, as they determine species’ regeneration strategies and biotic interactions [6]. Pollination modes, seed dispersal strategies, inflorescence types, and fruit morphology not only govern a species’ ability to reproduce successfully but also mediate its relationships with pollinators, dispersers, and other organisms [21,22]. Therefore, these traits capture unique dimensions of functional diversity that cannot be solely represented by vegetative traits, highlighting their necessity in integrated functional group classification.

As ecotones become increasingly vulnerable to climate shifts, understanding trait-based adaptation becomes urgent for conservation planning.

Located at the boundary between the mid-subtropical and southern subtropical zones, Niumulin Nature Reserve exhibits species characteristics of both evergreen broadleaf and subtropical forests [23]. This makes it an ideal site for studying trait-based functional group patterns in transitional ecosystems. Previous research in this area has primarily focused on single traits such as leaf morphology or reproductive phenology, leaving room for a more comprehensive analysis [24]. To address this, we systematically analyzed 17 vegetative and reproductive traits across 121 woody plant species in Niumulin Nature Reserve. Accordingly, this study aims to (1) conduct an integrated trait analysis of woody plants in a subtropical ecotone; (2) identify functional groups using hierarchical clustering methods; and (3) explore how trait syndromes reflect adaptive strategies to microhabitat variability.

## 2. Results

### 2.1. Vegetative Traits

**Figure 1:**
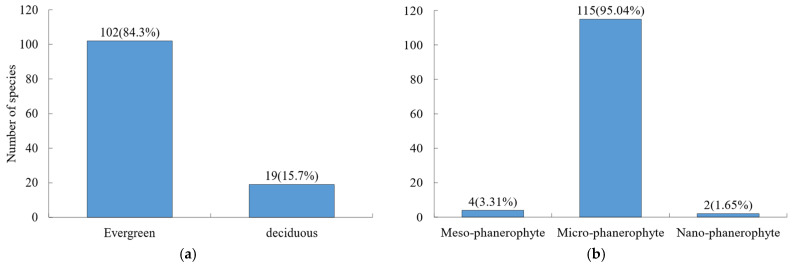
Composition of morphological traits for woody species. (**a**) Leaf habit; (**b**) life form. Values outside and inside parentheses indicate species number and percentage, respectively. The same is true below.

A total of 121 woody plant species were recorded in the Niumulin forest community, including 100 shrub species and 21 tree species. In terms of leaf habit (Figure 1a), evergreen species prevailed, with 102 species (84.3%), while deciduous species comprised 19 species (15.7%). Microphanerophytes were the dominant life form (Figure 1b), accounting for 95.04% of all species.

**Figure 2:**
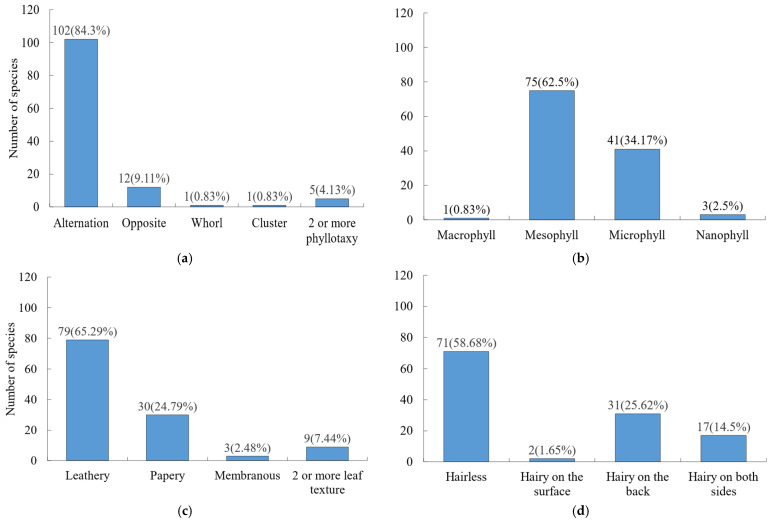
Frequency distribution of key leaf traits among woody plants. (**a**) Phyllotaxy; (**b**) leaf area; (**c**) leaf texture; (**d**) leaf indumentum.

Leaf morphology was dominated by simple leaves (113 species), with only 8 species bearing compound leaves. In terms of phyllotaxis (Figure 2a). Alternate phyllotaxis was most common, while opposite, whorled, and fascicled arrangements were less frequent. For leaf size (Figure 2b), medium or small sizes prevailed, and, for leaf texture (Figure 2c), leathery or papery textures were dominant. Entire leaf margins accounted for 60.33% of species, while non-entire margins represented 39.67%. Regarding leaf indumentum (Figure 2d), most leaves were glabrous (71 species), followed by those with hairs on the abaxial surface (31 species), and only 2 species had hairs on the adaxial surface.

### 2.2. Reproductive Traits

**Figure 3:**
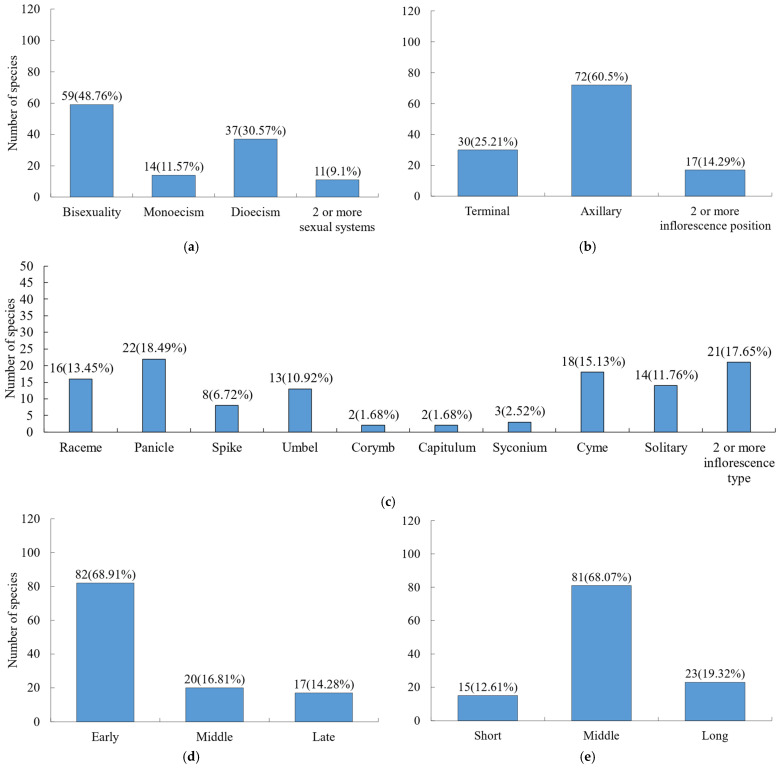
Composition of flower traits for woody plants. (**a**) Sexual system; (**b**) inflorescence position; (**c**) inflorescence type; (**d**) flowering stage; (**e**) flowering duration.

In terms of sexual systems (Figure 3a), hermaphroditic flowers dominated (48.76%), followed by dioecious species (30.57%). For inflorescences (Figure 3b,c), the position was primarily axillary, with panicles (18.49%), cymes (15.13%), racemes (13.45%), solitary flowers (11.76%), and umbels (10.92%) being the most common types. Regarding phenological observations (Figure 3d), most species exhibited early flowering, with 68.91% initiating flowering in the early growing season. Flowering duration (Figure 3e) was generally moderate across species.

**Figure 4:**
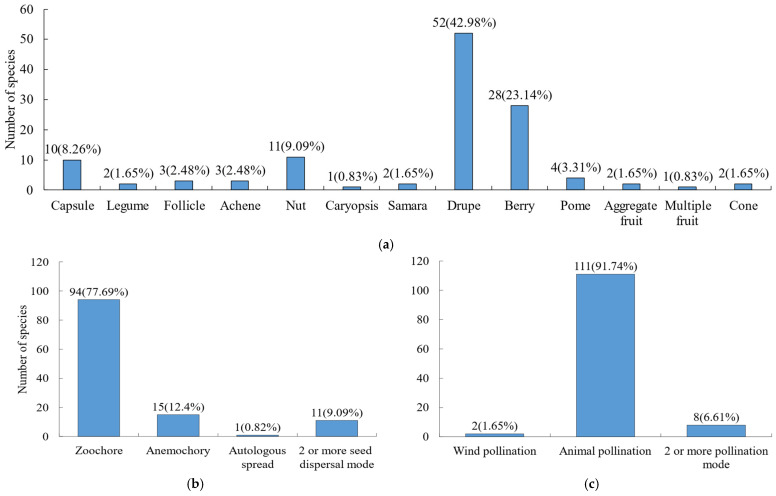
Fruit type, seed dispersal and pollination modes of woody plants. (**a**) Fruit type; (**b**) seed dispersal mode; (**c**) pollination mode.

For fruit types (Figure 4a), the most prevalent were drupes (42.98%) and berries (23.14%), followed by nuts (9.09%) and capsules (8.26%). Other fruit types such as legumes, follicles, and achenes were relatively rare. Both seed dispersal and pollination are important processes for plants to carry out sexual reproduction. Regarding pollination and seed dispersal strategies (Figure 4b,c), animal-mediated pollination and seed dispersal were the dominant reproductive strategies in the Niumulin community. Specifically, 91.74% of species relied on animals for pollination, and 77.69% for seed dispersal.

### 2.3. Classification and Characterization of Functional Groups

**Figure 5:**
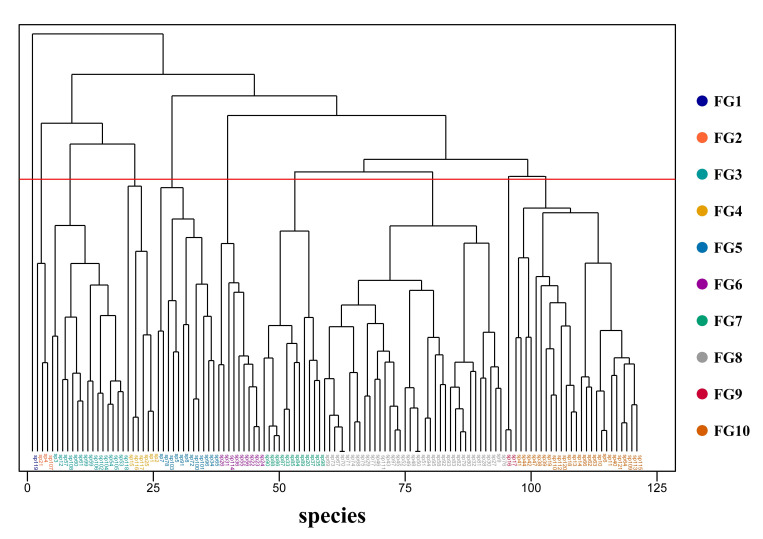
Dendrogram from the hierarchical clustering of 121 woody plant species based on 17 functional traits. Different colors represent the 10 identified functional groups, and the red line indicates the clustering threshold.

Hierarchical clustering of 17 functional traits across 121 woody species resulted in 10 distinct functional groups (FGs), each characterized by unique combinations of vegetative and reproductive traits (Figure 5, Table S2).

FG1: A unique group contained only *Meliosma rigida* Siebold & Zucc., distinguished by its whorled phyllotaxy and large-sized leaves. This singleton group possessed a unique functional syndrome—the combination of whorled phyllotaxy and large leaves, which was not found in any of the other 120 woody plant species in the region. This trait combination corresponded to a specialized adaptive strategy for high-light microhabitats (e.g., forest gaps, edges), and its ecological uniqueness had been verified through field microhabitat surveys, which made it not an outlier of clustering.

FG2: This group comprised mesophanerophyte evergreen trees, with propagule structures of cones or caryopses, and an early flowering period. The species included *Cunninghamia lanceolata* (Lamb.) Hook., *Phyllostachys edulis* (Carrière) J. Houzeau, and *Pinus massoniana* Lamb.

FG3: This group was hermaphroditic, microphanerophytes with alternate leaves, relying on animals for pollination. The woody plants included species from Rosaceae, Theaceae, Fabaceae, and others, including *Anneslea fragrans* Wall., *Photinia prunifolia* (Hook. et Arn.) Lindl., *Rhaphiolepis ferruginea* F. P. Metcalf, *Rhaphiolepis ferruginea* var. *serrata, Itea omeiensis* C. K. Schneid., *Michelia odora* (Chun) Noot. & B. L. Chen, *Sloanea sinensis* (Hance) Hemsl., *Archidendron lucidum* (Benth.) I. C. Nielsen, and *Malus doumeri* (Bois) A. Chev.

FG4: This group was dominated by rare species, all of which were microphanerophyte shrubs with terminal inflorescences, that primarily relied on wind for pollination or seed dispersal. The woody plants comprised *Acer cordatum* Pax, *Acer tutcheri* Duthie, *Engelhardia roxburghiana* Wall., *Metadina trichotoma* (Zoll. & Moritzi) Bakh. f., *Tetradium glabrifolium* (Champ. ex Benth.) T. G. Hartley, and *Tetradium austrosinense* (Hand-Mazz.) T. G. Hartley. The existence of this small FG reflected the fine niche differentiation in the ecotone. These species shared the core strategy of wind-dependent pollination/dispersal, adapted to the complex wind environment in mountainous areas, and formed a stable functional syndrome rather than random clustering results.

FG5: This group was mainly composed of deciduous tree species, all with alternate leaves, axillary inflorescences, and animal pollination. The woody plants consisted of *Nyssa sinensis* Oliv., *Choerospondias axillaris* (Roxb.) B. L. Burtt & A. W. Hill, *Ilex asprella* (Hook. & Arn.) Champ. ex Benth., Diospyros tsangii Merr., *Antidesma japonicum* Sieb. et Zucc., *Ilex macrocarpa* Oliv., *Diospyros kaki* var. *sylvestris* Makino, *Toxicodendron succedaneum* (L.) Kuntze, *Lindera glauca* (Siebold & Zucc.) Blume, *Ficus heteromorpha* Hemsl., *Ficus hirta* Vahl, and *Liquidambar formosana* Hance.

FG6: Evergreen microphanerophyte shrubs with simple and entire leaves, distinguished by opposite phyllotaxis—a unique trait among the four functionally overlapping groups—and strictly entire leaves with no exceptions. The woody plants included species of *Gardenia jasminoides* J. Ellis, *Aidia cochinchinensis* Lour., *Diplospora dubia* (Lindl.) Masam., *Neolitsea aurata* (Hayata) Koidz., *Symplocos glauca* (Thunb.) Koidz., *Euonymus laxiflorus* Champ. & Benth., *Chengiodendron matsumuranum* (Hayata) C. B. Shang, X. R. Wang, Yi F. Duan & Yong F. Li, *Tarenna mollissima* (Hook. & Arn.) B. L. Rob., and *Syzygium buxifolium* Hook. et Arn.

FG7: This group was typically evergreen trees with nuts, simple alternate leaves, dependent on animals for pollination and seed dispersal. The woody plants consisted of *Ficus variolosa* Lindl. ex Benth., *Fissistigma uonicum* (Dunn) Merr., *Artocarpus hypargyreus* Hance, *Quercus glauca* Thunb., *Michelia maudiae* Dunn, and species from the genus *Castanopsis* (Fagaceae).

FG8: Evergreen microphanerophytes with simple alternate leaves, relying on animal-mediated pollination and dispersal. Its notable features are axillary inflorescences (mainly panicles or cymes) and papery leaf texture, with most species being understory generalists adaptable to low-light environments. The woody plants included species of *Diospyros morrisiana* Hance, *Maesa japonica* (Thunb.) Moritzi, *Myrsine seguinii* H. Lév., *Trema tomentosa* (Roxb.) Hara, *Trema cannabina* Lour., and most species from Aquifoliaceae, Symplocaceae, and Lauraceae.

FG9: Evergreen microphanerophyte shrubs with simple leaves, characterized by the unique combination of medium-sized leaves, leathery texture, and glabrous surfaces—traits that collectively distinguish it from the other three groups. It included *Machilus chekiangensis* S. Lee and *Ilex qingyuanensis* C. Z. Zheng.

FG10: Evergreen microphanerophyte shrubs with alternate leaves, pollinated and their seeds dispersed by animals. It differs from the other three groups in its diverse fruit types (coexisting drupes, berries, and nuts) and longer flowering periods, with some species capable of adapting to both understory and forest-edge microhabitats. It includes species of *Mallotus lianus* Croizat, *Lithocarpus oleifolius* A. Camus, *Machilus grijsii* Hance, *Machilus thunbergii* Sieb. et Zucc., *Machilus pauhoi* Kaneh., *Ilex kwangtungensis* Merr., *Symplocos fukienensis* Y. Ling, *Symplocos congesta* Benth., *Symplocos theophrastifolia* Siebold & Zucc., *Pygeum topengii* Merr., *Helicia cochinchinensis* Lour., *Cryptocarya chingii* W. C. Cheng, *Phoebe chekiangensis* C. B. Shang, *Meliosma squamulata* Hance, *Meliosma thorelii* Lecomte, *Elaeocarpus decipiens* Hemsl., *Cinnamomum subavenium* Miq., *Cinnamomum austrosinense* Hung T. Chang, *Alangium chinense* (Lour.) Harms, *Cinnamomum micranthum* (Hayata) Hayata, etc.

## 3. Discussion

By integrating key vegetative and reproductive traits, we identified distinct functional groups of woody plants in a mid-subtropical–south-subtropical ecotone. These groups encapsulate the core adaptive strategies of species and reveal the main drivers of community assembly, advancing our understanding of how woody plants coexist in transitional environments. Below, we discuss these findings in the context of trait syndromes, niche differentiation, and conservation implications.

### 3.1. Integrated Trait Syndromes and Ecotonal Filtering Drive Functional Group Assembly

Our analysis demonstrates that the assembly of woody plants in the Niumulin ecotone is shaped by the integration of vegetative and reproductive traits, a pattern consistent with strong environmental filtering and adaptive trade-offs. It should be noted that all trait frequency statistics in this study are species-weighted (based on the proportion of species number rather than individual abundance). This design is closely related to the core goal of the study—we focus on the trait differentiation patterns and assembly mechanisms of functional groups, rather than the functional contribution of single dominant species. Species weighting can more comprehensively cover the unique functional traits of rare species, avoiding the masking of functional diversity in key dimensions by abundance differences (e.g., the wind-dependent strategy of rare species in FG4). The dominance of evergreen microphanerophytes (84.3%; 95.04%) establishes a fundamental vegetative structure that facilitates resource partitioning along vertical and temporal gradients, which adapted to regional ecological conditions [25,26]. Deciduous species in the canopy layer (e.g., FG5) capitalize on spring light resources through rapid leaf expansion, while evergreen microphanerophytes in the understory (e.g., FG6, FG8) maintain year-round carbon assimilation in low-light conditions [27,28,29]. This stratification is a hallmark of mixed forests in transitional zones, where niche complementarity enhances community-level resource use efficiency [30].

The prevalent leaf economic spectrum—simple, medium-sized, leathery leaves with entire margins—reflects a functional strategy compatible with the warm, humid subtropical climate, in line with the inference of adaptive optimization. Simple leaves reduce construction costs and improve hydraulic efficiency [4], while leathery textures with thick cuticles confer resistance to high irradiance and reduce water loss [31,32]. The notably high proportion of entire margins in Niumulin, compared to core mid-subtropical forests, may be attributed to milder winter temperatures in this ecotone, reducing selective pressure for serrated margins that can mitigate cold-induced photodamage [33]—a correlative explanation consistent with environmental filtering. Phyllotaxy further fine-tunes light capture: alternate leaves minimize self-shading in the understory [34], whereas the opposite phyllotaxy in FG6 suggests a compact crown architecture suited to specific low-light microsites [35,36]. The unique combination of whorled phyllotaxy and large leaves in FG1 represents a specialized strategy for high-light microhabitats, maximizing light interception while minimizing mutual shading, consistent with patterns observed at forest edges [37]—a trait pattern that aligns with the inference of adaptive specialization.

Critically, reproductive traits were assembled in parallel with vegetative strategies, underscoring the necessity of an integrated trait perspective. The dominance of early flowering and axillary inflorescences likely enhances reproductive stability in a hot, rainy climate by protecting floral organs and ensuring pollination success [38,39]. In this study, 68.91% of species concentrated their flowering in the early growing season (April–June) with moderate flowering duration. This pattern of synchronized reproductive timing is presumably closely related to the seasonal characteristics of the subtropical monsoon climate in this region: the early growing season is characterized by suitable temperatures (average 18–25 °C) and abundant, evenly distributed precipitation, which not only avoids the adverse effects of high temperature and heavy rain on pollen viability and pollination but also provides stable resource supply for pollinators, thereby improving reproductive success [40]. The potential association between such climatic signals and reproductive synchrony reflects the adaptive strategies of plants in the ecotone to environmental fluctuations, and also confirms the important role of climatic filtering in the assembly of reproductive traits. The overwhelming reliance on animal-mediated pollination (91.74%) and seed dispersal (77.69%), coupled with the prevalence of drupes and berries, highlights the ecosystem’s profound dependence on faunal mutualists. This aligns with the global observation that plant–animal interactions are pivotal for maintaining the regeneration and stability of species-rich forests [5,41]. The co-occurrence of these vegetative and reproductive traits in our functional groups demonstrates that plants in this ecotone coordinate multiple trait dimensions to cope with environmental heterogeneity, a pattern consistent with integrated adaptive strategies that is often overlooked in single-trait studies [11,12].

### 3.2. Functional Group Classification Reveals Niche Differentiation and Adaptive Strategies

The hierarchical clustering of 121 species into 10 distinct FGs synthesizes multi-trait trade-offs, providing a mechanistic understanding of community assembly in the ecotone—with trait differentiation among groups as a demonstrated pattern, and niche-based processes as an inferred mechanism supporting community structure. Each FG represents a unique ecological strategy, and the clear differentiation among them strongly supports the role of niche-based processes in structuring this community. It is important to clarify that the functional groups (FGs) in this study are empirical groupings based on the convergence of core functional trait syndromes, rather than rigid ecological units with absolutely clear and non-overlapping boundaries. There is overlap in some basic traits among groups (e.g., most groups share the “simple leaf” trait), which is a normal phenomenon in the process of ecological strategy convergence and does not affect the identification of core functional differences; at the same time, there is slight trait variability within groups [42]. Such variability is a supplementary adaptation of species to microhabitat heterogeneity, further reflecting the integrity of community functional diversity.

FG2, comprising mesophanerophyte evergreen trees like *Cunninghamia lanceolata* and *Pinus massoniana* with cones/caryopses and early flowering, exemplifies a strategy consistent with pioneer adaptation to exploit canopy gaps and early-season resource availability [43]. In contrast, FG5, dominated by deciduous trees (e.g., *Nyssa sinensis*, *Liquidambar formosana*), employs a deciduous habit to minimize carbon loss during the unfavorable season, a trait pattern well-documented as a potential response to environmental stress in transitional forests [44]. The functional groups also illuminate the strategies of understory specialists. FG6 and FG8, both composed of evergreen microphanerophytes, achieve persistence through different means: FG6 utilizes opposite phyllotaxy and entire leaves for compact light harvesting, while FG8 relies on animal-mediated dispersal for recruitment in shaded conditions. This demonstrates how divergent trait syndromes can facilitate coexistence within the same forest stratum by reducing interspecific competition [41]—an observed pattern that infers niche differentiation.

Notably, the classification crystallized the strategies of rare species, which are often key to functional diversity. Regarding FG4, which includes rare species like *Acer cordatum*, its trait of wind-dependent reproduction (terminal inflorescences, anemochory) is speculated to be a potential adaptive strategy for the complex wind patterns in mountainous terrain—consistent with the theory that rare species often occupy narrow functional niches [45]—but this hypothesis requires further verification through long-term targeted monitoring. Conversely, no flowering traits have been recorded for FG9 (*Machilus chekiangensis*, *Ilex qingyuanensis*) so far. This phenomenon may imply a potential reliance on cryptic sexual reproduction or vegetative propagation, or it could be affected by factors such as limited sampling duration or phenological mismatch; no definitive conclusion can be drawn solely based on existing observations. This reproductive uncertainty urgently needs to be clarified through targeted research such as long-term phenological tracking and population dynamics monitoring. The unique functional identity of FG1 (*Meliosma rigida*) further highlights how ecotones can harbor distinct functional strategies adapted to specific microhabitats, contributing to overall ecosystem complexity [37]. In addition, both the adaptive strategies of rare species in FG4 and the reproductive mode of FG9 require targeted sampling with an extended sampling period covering more microhabitats, as well as long-term research such as annual continuous phenological observations, to improve the reliability of conclusions. This also points out the direction for in-depth research on plant reproductive strategies in this region.

### 3.3. Implications for Biodiversity Conservation in an Ecotone Forest

The functional group classification in this study is based on presence–absence data rather than abundance- or dominance-weighted data, a choice justified by the research objectives and the inherent characteristics of the study area, for the following reasons. First, the core goal of this study is to reveal the assembly mechanisms and trait differentiation patterns of functional groups, and the core definition of functional groups lies in “the convergence of species functional trait syndromes” [46]. Presence–absence data can fully capture such cross-species functional differences and accurately reflect the convergence and differentiation of ecological strategies. Second, as a mid-subtropical–south-subtropical ecotone, the study area has complex terrain and high microhabitat heterogeneity. Some species (e.g., rare species in FG4) possess irreplaceable functional traits (e.g., wind-dependent pollination/dispersal) despite low abundance. Presence–absence data ensures the inclusion of these species in the classification, providing comprehensive coverage of the community’s functional diversity, which is an objectively necessary choice to guarantee the completeness of functional group classification. In addition, the subsequent discussions on conservation and ecosystem function in this study focus on the “trait complementarity” of functional groups—different functional groups jointly maintain ecosystem stability through unique adaptive strategies. The realization of this complementarity relies on the complete coverage of functional traits, rather than the abundance advantage of a single species, which is highly consistent with the analytical logic of presence–absence data [47].

The functional group differentiation observed in Niumulin provides a robust, trait-based framework for predicting ecosystem responses to environmental change and informing conservation practice. The heavy reliance on animal mutualists, evident in FGs 3, 7, 8, and 10, implies that the conservation of pollinator and disperser communities is paramount for maintaining the reproductive resilience of this forest [5,41]. Any decline in these animal populations could trigger cascading effects, disproportionately affecting a large portion of the woody plant community.

Furthermore, the identification of distinct FGs allows for targeted conservation strategies. The preservation of rare species in FG4 requires maintaining the specific microhabitat conditions and wind dynamics that facilitate their wind-based reproductive strategy [45]. The unique functional identity of FG1 highlights the conservation value of high-light microhabitats, such as forest edges or gaps, which may be critical for maintaining overall functional diversity [37]. The potential reliance on cryptic reproduction in FG9 underscores the need for detailed autecological studies to fully understand the life history and vulnerabilities of seemingly common species.

In conclusion, our integrated classification, by considering both vegetative and reproductive traits, has proven powerful in deciphering the assembly rules of this ecotonal forest. It reveals that species coexistence is facilitated by a division of labor among functionally distinct groups, each adapted to specific niches within the heterogeneous landscape. Due to the lack of direct environmental measurement data, the depth of mechanistic inference between functional groups and environmental filtering in this study is limited. In the future, quantitative correlation analysis between functional traits and environmental variables can be conducted by synchronously measuring key environmental factors such as light, soil moisture, and temperature to further verify the driving effect of environmental filtering. As ecotones like Niumulin face increasing pressure from climate change and anthropogenic disturbance, this trait-based approach offers a predictive framework for prioritizing conservation efforts. Future work should focus on linking these functional groups to ecosystem processes (e.g., carbon sequestration, nutrient cycling) and monitoring their temporal dynamics to manage these transitional forests for both biodiversity and functional resilience [30,48].

## 4. Materials and Methods

### 4.1. Study Area

This study was conducted in the Niumulin Nature Reserve (25°21′–25°28′ N, 118°07′–118°15′ E), located in Southeast China. The reserve lies on the southeastern slope of the Daiyun Mountains and encompasses a typical ecotone between the mid-subtropical and south-subtropical climatic zones. Characterized by a subtropical monsoon climate [49], the area features complex topography with elevations ranging from 200 to 1000 m above sea level, creating a gradient from low hills to mid-mountain terrain.

The vegetation exhibits distinct transitional characteristics, incorporating floristic elements from both subtropical evergreen broad-leaved forests and tropical seasonal rainforests [23]. The unique environment of this ecotone, such as seasonal drought and temperature fluctuations, are expected to drive the formation of distinct plant strategies. This complex interplay of environment and biodiversity makes the area an ideal system for investigating the integration of functional traits and the differentiation of functional groups.

### 4.2. Plot Design and Sampling

To capture the spatial heterogeneity of terrain and vegetation, a combination of systematic and random sampling was employed. A total of 19 plots (40 m × 40 m each) were established along an altitudinal gradient in the core area of the reserve, with 1–4 adjacent plots per elevation band. The selection of altitudinal gradient was intended to cover the significant microhabitat heterogeneity mediated by topographic variation—e.g., higher humidity and milder light conditions in low-altitude areas, and more complex wind environments with greater temperature fluctuations in high-altitude areas. This design ensures that the collected woody plant species fully represent the functional trait diversity of the ecotone, providing a representative species pool for subsequent functional group classification. Each plot was georeferenced using Beidou handheld GPS receiver (G128BD; UniStrong, Beijing, China), and topographic variables including elevation, slope gradient, and slope aspect were recorded.

Each plot was subdivided into four 20 m × 20 m subplots. Corners were marked with PVC pipes and delineated using nylon ropes to ensure clear and traceable boundaries. Within each subplot, all woody plant species with a diameter at breast height (DBH) ≥ 1 cm within these subplots were identified and tagged, and their DBH was measured.

### 4.3. Functional Trait Data Collection

We measured 17 key functional traits (for a complete list including definitions, see Supplementary Table S1), encompassing vegetative, architectural, and reproductive dimensions.

Species classification criteria: Shrubs and trees were strictly classified based on tree height data, which was calculated as the average after excluding abnormal extreme values in each plot to effectively avoid data bias. The sampling design of this study (40 m × 40 m plots + DBH ≥ 1 cm inclusion criterion) did not favor shrubs, and the distribution pattern of 100 shrub species and 21 tree species truly reflects the inherent community characteristics under the heterogeneous microhabitats of the mid-subtropical–south-subtropical ecotone.

Species identification: Species identification was based on *Flora of China* and *Flora of Fujian* [50,51]. For uncertain taxa, specimens (flowering and fruiting branches) were collected and verified by experts at the *Herbarium of Fujian Agriculture and Forestry University*.

Vegetative Traits: DBH was measured using tree diameter tape (2 m, ±0.1 cm; Wintape, Foshan, China), and tree height was measured using an ultrasonic laser altimeter (Vertex 5, ±0.15 m; Haglof Sweden AB, Långsele, Sweden). For low-stature shrubs, height was measured directly with a tape. From each individual, 3–5 healthy branches were selected from the upper-middle canopy, and three mature, undamaged leaves were collected per branch (avoiding young or senescent leaves). Leaf area was determined from scanned images using a mobile application for leaf area and herbivory measurement (LeafByte, iOS version 1.4.0; Cornell University, Ithaca, NY, USA), and leaf thickness was measured at the lamina adjacent to the main vein using a digital caliper (293-230-30, ±0.01 mm; Mitutoyo Corporation, Kawasaki, Japan). Qualitative traits such as pubescence and leaf margin were recorded with the aid of a stereomicroscope (SMZ-168, 10× magnification, 7.5×–50× total magnification range; Motic Corporation, Xiamen, China).

Reproductive Traits: To account for habitat heterogeneity, phenological observations were conducted across different microhabitats. For each species, healthy adult individuals were randomly selected and georeferenced. Observations were carried out at regular intervals throughout the year, and key phenological phases (leaf flushing, flowering, fruiting) were recorded [52]. The first and last occurrence dates of each phase were recorded by field notes. For the phenological types and flowering and fruiting periods of some rare plants, refer to *Flora of Fujian* and *Flora of China* [50,51]. Inflorescences and fruits were collected during the flowering and fruiting seasons, respectively. Inflorescence type, position, and floral characteristics were recorded. Diaspore type was determined based on fruit and seed morphology. Seed dispersal vectors were determined through a combination of direct field observations of animal feeding behavior and inference based on diaspore morphology. Data on pollination and seed dispersal modes were mainly derived from the authoritative literature such as *Flora of China* and *Flora of Fujian* [50,51]. The classification criteria referred to the typical recorded pollination/dispersal modes of plants in the same family and genus to ensure consistent classification logic: for species with mixed strategies (e.g., some species with both animal and wind dispersal characteristics), they were uniformly classified into the “2 or more” category (corresponding to “2 or more pollination mode” and “2 or more seed dispersal mode” in the results); for species with ambiguous literature records, supplementary verification was conducted by consulting the trait characteristics of their closely related species and more regional floras to finally confirm the classification [50,51].

### 4.4. Statistical Analysis and Functional Group Classification

Trait weights were adjusted to balance the influence of different trait categories. All statistical analyses were performed in R software (v4.2.2).

It should be clarified that topographic variables (elevation, slope gradient, aspect) were not incorporated into the quantitative analysis of functional group classification. The hierarchical clustering solely relied on the 17 key vegetative and reproductive functional traits, focusing on revealing the convergence of species’ adaptive trait syndromes.

All traits were standardized and categorized following explicit established protocols to ensure consistency and comparability across species—specifically referencing internationally recognized frameworks [48], technical specifications for Chinese forest biodiversity monitoring, and data standards from the global TRY Plant Trait Database, aligned with research processes [46]. The processing methods for different trait types are as follows: (1) Continuous traits (e.g., leaf area, DBH): Z-score standardization (z = (x − μ)/σ) to eliminate unit effects and balance trait weights. (2) Categorical traits (e.g., inflorescence type, pollination mode): One-hot encoding into binary dummy variables (0 = absent, 1 = present) to adapt to mixed-type data clustering. (3) Semi-quantitative traits (e.g., leaf hairiness density): Ordinal classification (1–3 grades) based on authoritative floras and field standards, then converted to numerical values.

Strict quality control was implemented: trait values deviating > 3 standard deviations were verified against original records and corrected or excluded. All traits were integrated using the ‘vegan’ (trait data integration) package in R.

Gower’s distance was used to calculate pairwise species dissimilarities, as it accommodates mixed data types [53]. Hierarchical clustering was performed via Ward’s minimum variance method to maximize inter-group dissimilarity, with cluster stability assessed by 1000 bootstrap resamplings (implemented in the ‘pvclust’ (cluster stability assessment) package). The optimal number of functional groups (10 groups) was determined by combining the Elbow Method (quantitative indicator) and ecological interpretability [46], ensuring a balance between compactness, separation, and ecological significance.

## 5. Conclusions

We integrated 17 vegetative and reproductive functional traits across 121 woody plant species, successfully classifying the community into 10 distinct functional groups (FGs) through hierarchical clustering. Our analysis reveals the key trait differentiation patterns and assembly mechanisms of woody plants in the critical mid-subtropical–south-subtropical ecotone. The characteristic integration of these FGs—such as vertical stratification mediated by leaf phenology, the optimization of light capture through leaf trait diversification, and the dominance of animal-mediated pollination and seed dispersal—collectively illustrate a synergistic adaptation to the complex microhabitats of this transitional zone. These findings provide a vital theoretical basis for understanding community assembly, refining biodiversity conservation strategies, and predicting ecosystem functionality in subtropical forests under environmental change. While providing a foundational classification, this study has limitations in geographic representativeness, mechanistic trait representation, and temporal dynamics, which are critical to address in future research.

## Data Availability

The raw data supporting the conclusions of this article will be made available by the authors on request.

## Supplementary Materials

The following supporting information can be downloaded at: https://www.mdpi.com/article/10.3390/plants15030406/s1, Table S1 [54,55,56]: The Classification of Vegetative and reproductive Traits; Table S2: Functional groups.
