# Supplementary material for: Integrated Vegetative and Reproductive Traits Reveal Functional Groups and Assembly Mechanisms in a Subtropical Forest Ecotone
Source: Plants (Basel). 2026 Jan 29;15(3):406. doi: 10.3390/plants15030406 (PMC12899091; doi:10.3390/plants15030406)
Supplement: Supplementary file 1 [file plants-15-00406-s001.zip › Table S1.pdf]

Supporting Information

Table S1: The Classification of Vegetative and reproductive Traits [54-56]

Functional trait	Style	Classification criteria
Growth form	Arbor	Tree height ≥ 5m
	Shrub	Tree height＜5m
Life form	Meso-phanerophyte	Tree height ranges from 8 to 30 meters
	Micro-phanerophyte	Tree height ranges from 2 to 8 meters
	Nano-phanerophyte	Tree height ranges from 0.25 to 2 meters
Leaf life cycle	Evergreen leaf	Maintain green leaves throughout the year
	Fallen leaf	Leaves fall off intensively at the end of a specific growing season
Leaf type	Simple leaf	A single leaf blade is borne on a petiole
	Compound leaf	Multiple leaflets are borne on a single petiole
Phyllotaxy	Alternation	One leaf is borne at each node, arranged alternately
	Opposite	Two leaves are borne at each node, arranged oppositely
	Whorl	Three or more leaves are borne at each node, arranged in a whorl
	Cluster	Multiple leaves are borne on shortened stem nodes in a dense cluster
	2 or more phyllotaxy	Different phyllotaxes exist
Leaf area	Macrophyll	Leaf area ranges from 182.25 to 1642.25 cm^2
	Mesophyll	Leaf area ranges from 20.25 to 182.25 cm^2
	Microphyll	Leaf area ranges from 2.25 to 20.25 cm^2
	Nanophyll	Leaf area ranges from 0.25 to 2.25 cm^2
Leaf texture	Leathery	The leaf texture is dense and tough
	Papery	The leaf texture is relatively thin and flexible
	Membranous	The leaf texture is extremely thin
	2 or more leaf texture	Different leaf textures exist
Leaf margin	Entire leaf	The leaf margin is continuous and smooth
	Non-entire leaf	The leaf margin is discontinuous
Leaf indumentum	Hairless	There are no visible trichomes on the leaf blade
	Hairy on the surface	Trichomes are present only on the surface of the leaf blade
	Hairy on the back	Trichomes are present only on the abaxial surface of the leaf blade
	Hairy on both sides	Trichomes are present on both surfaces of the leaf blade
Sexual system	Bisexuality	A flower with both stamens and pistils

	Monoecism	Both male and female flowers are borne on the same plant
	Dioecism	Male and female flowers are borne on separate plants
	2 or more sexual systems	Different sexual systems exist
Inflorescence type	Raceme	Numerous flowers with nearly equal-length pedicels are borne on the rachis
	Panicle	The rachis is branched, with each branch bearing a simple inflorescence
	Spike	Numerous sessile or subsessile flowers are borne on the rachis
	Umbel	Numerous flowers with nearly equal-length pedicels are borne at the apex of the rachis and arranged in an umbellate shape
	Corymb	Flowers on rachis with unequal pedicels, lower longer, arranged in nearly same plane
	Capitulum	Shortened discoid/convex rachis with dense sessile flowers
	Syconium	Closed saccate rachis enclosing flowers
	Cyme	Determinate, terminal flower first, successive lateral apex flowers
	Solitary	Single on stem or branch
	2 or more inflorescence type	Different inflorescence type exist
Inflorescence position	Terminal	Borne at stem/branch apex
	Axillary	Borne in leaf axils
	2 or more inflorescence position	Different inflorescence position exist
Propagule structure	Capsule	Dry syncarpous fruit dehiscent at maturity
	Legume	Monocarpellary, dehiscent along both sutures
	Follicle	Monocarpellary fruit dehiscent along one suture
	Achene	Dry, indehiscent fruit with single seed and pericarp free from seed coat
	Nut	Dry, indehiscent fruit with hard pericarp and single seed
	Caryopsis	Dry, indehiscent, single-seeded, pericarp fused with seed coat
	Samara	Dry, indehiscent fruit with winged pericarp
	Drupe	Fleshy fruit with hard endocarp (stone) enclosing seed
	Berry	Fleshy fruit with succulent pericarp and many seeds
	Pome	Fleshy pseudocarp from ovary and receptacle

	Aggregate fruit	Fruitlets from multiple apocarpous carpels of a single flower, aggregated on receptacle
	Multiple fruit	Derived from an entire inflorescence with multiple floral parts
	Cone	gymnosperm structure with woody scales bearing seeds
Seed dispersal mode	Zoochore	Seed dispersal via animal mediation
	Anemochory	Seed dispersal via wind
	Autologous spread	Seed dispersal via plant's own mechanisms
	2 or more seed dispersal mode	Different seed dispersal modes exist
Pollination mode	Wind pollination	Pollination via wind
	Animal pollination	Pollination via animals
	2 or more pollination mode	Different pollination modes exist
Flowering stage	Early	Flowering period occurs between January and May
	Middle	Flowering period occurs between June and July
	Late	Flowering period occurs between August and December
Flowering duration	Short	Flowering duration is within one month
	Middle	Flowering duration is 2–3 months
	Long	Flowering duration is four months or longer

References:

54. Yuan, Q.; Cao, J.Y.; Liu, J.F.; Xu, D.Y.; Fan, H.D.; Tan, B.; Xu, D.N.; Liu, L.B.; Ye, D.; Ni, J. Statistical bias of plant functional traits in forest ecosystems caused by different classifications of growth form. Acta Ecologica Sinica 2021, 41(3): 1106-1115. DOI: 10.5846/stxb202002010188.
55. Ma, S.; Li, D. Dispersal and Evolution in Higher Plants Ⅰ.Diaspores, Their Quantity and Life Span as well as Dispersal Mechanisms. Acta Botanica Yunnanica 2002, 24(005):569-582. DOI: 10.3969/j.issn.2095-0845.2002.05.003.
56. Zou, L.; He, Z; Jiang L; Liu J; Zheng S; Li W. Pollination Mode of Spermatophyte in Daiyun Mountain Nature Reserve. Chinese Agricultural Science Bulletin 2017, 33(4): 38-42 https://doi.org/10.11924/j.issn.1000-6850.casb16050105.
